# A mathematical model to study resistance and tolerance to infection at the animal and population levels: application to *E. coli* mastitis

**DOI:** 10.3389/fgene.2012.00146

**Published:** 2012-12-14

**Authors:** Johann C. Detilleux

**Affiliations:** Quantitative Genetics Group, Faculty of Veterinary Medicine, University of LiègeLiège, Belgium

**Keywords:** resistance, tolerance, infection, mathematics

## Abstract

A mathematical model is proposed that describes the colonization of host tissues by a contagious pathogen and the early nonspecific immune response, the impact of the infection on the performances of the host, and the spread of the infection in the population. The model obeys specific biological characteristics: Susceptible hosts are infected after contact with an infected one. The number of pathogenic units that invade a susceptible host is dependent on the infectious dose provided by the infected host and on the ability of the susceptible host to resist the invasion. After entry in host, pathogenic changes over time are expressed as the difference between the intrinsic logistic growth rate and the Holling type II kill rate provided by the immune response cells. Hosts have different ability to restrict reproduction of the pathogen units. The number of response cells actively recruited to the site of infection depends on the number of the pathogenic units. Response cells are removed after having killed a fixed number of pathogenic units. The effects of the number of pathogenic units on the performances of the host depend upon its levels of tolerance to the deleterious effects of both pathogenic and response cells. Pre-infection costs are associated to tolerance and resistance levels. Estimates of most biological parameters of the model are based on published experimental studies while resistance/tolerance parameters are varied across their allowable ranges. The model reproduces qualitatively realistic outcomes in response to infection: healthy response, recurrent infection, persistent infectious and non-infectious inflammation, and severe immunodeficiency. Evolution across time at the animal and population levels is presented. Effects on animal performances are discussed with respect to changes in resistance/tolerance parameters and selection strategies are suggested.

## Introduction

Many conservation and selection programs (e.g., FAO, 2007; Eadgene, 2012) include increasing ability to fight endemic disease as an objective. The first challenge to meet this objective is to accurately define and measure disease resistance and tolerance.

Resistance traits are broadly defined as host traits that reduce the extent of pathogen infection. They include traits that reduce pathogen transmission at contact and pathogen growth rate once infection has occurred (Kover and Schaal, 2002). Controlled immune response is a major mediator of resistance because of its efficacy in clearing infections (Sears et al., 2011). Operationally, resistance is typically measured as the inverse of infection intensity (number of parasites per host or per unit host tissue) and a lower intensity means an animal is more resistant, all else being equal (Råberg et al., 2009; Medzhitov et al., 2012).

Tolerance, on the other hand, is defined as the host's ability to reduce the effect of infection on its fitness. Although fitness measurements include different life-history traits, only performance (e.g., growth, milk or wool) is considered as it is very important in farm animals. Tolerance may be targeted to reduce damage directly inflicted by the pathogen (direct tolerance) or caused by the immune response (indirect tolerance). Little is known about underlying mechanisms of tolerance (see one example in Medzhitov, 2009) although they potentially include tissue repair and immunological mechanisms (Råberg et al., 2007). Tolerance is usually operationally defined as the slope of a regression of host performance against infection intensity and a steeper the slope means lower tolerance (Råberg et al., 2009; Medzhitov et al., 2012).

Costs are associated with both resistance and tolerance because energy is required to maintain immune-competence and to mount an efficient immune response, as shown in various empirical studies (Boots and Bowers, 1999; Canale and Henry, 2010). Microarray analyses of the early response to infection with mammary pathogens have also revealed reorganization of gene expression involved in energy metabolism (Bonnefont et al., 2012). If energy is required to uphold resistance and tolerance, less is available to maintain fitness (resource allocation theory; Oltenacu and Algers, 2005). So, authors have proposed to measure these resource allocation costs by comparing performances of resistant and tolerant hosts in pathogen-free environments (e.g., Nunez-Farfan et al., 2007; Rohr et al., 2010).

Unfortunately, levels and costs associated to resistance and tolerance are usually difficult to obtain in field studies under pathogen attack. Given these technical difficulties, their relative importance is here investigated using mathematical simulation studies. Hence, the main objective of the paper is to investigate and the effects of resistance and tolerance on the spread of an infectious disease and on the performances of the animals within a closed population, for a range of realistic scenarios. At the animal level, a comprehensive model is constructed that incorporates important biological characteristics associated with the early immune response to infection.

## Materials and methods

The model has two main components, each with two parts. The first system of equations describes the changes in cell concentrations associated with the infection. The second expresses the effects of the infection on host performances. Both are made stochastic rather than deterministic to capture the variability inherent in biological processes.

### System of equations for pathogen and immune cells dynamics

The system of equations elaborates on a previous discrete susceptible–infected–susceptible model (Detilleux, 2011) that considers a homogeneous population of size N in which a disease is spreading. Transmission of the disease occurs via direct animal–to–animal contact. Once infected, hosts are able to transmit the infection and are able to be re-infected. The infectious dose is assumed to depend on the pathogen burden in the infected host and the resistance of the susceptible one. After infection, pathogens multiply in the tissue environment and an innate immune response is mounted against them. In the absence of infection, immune effectors (called “response cells” throughout the manuscript) cycle throughout the body. During the early immune response, these response cells are recruited actively to the site of infection. Once they reach the site, they are activated and begin their task of digesting and destroying the invading pathogens.

For one individual, the within-host model follows the dynamics of response cells and pathogen populations:

(1) $$\begin{array}{l} B_{t+\Delta t}=B_{t}+D_{t+\Delta t}+N_{t+\Delta t}-K_{t+\Delta t}\\ C_{t+\Delta t}=C_{t}+M_{t+\Delta t}+G_{t+\Delta t}-S_{t+\Delta t} \end{array}$$

where *B*_*t*_ is the concentration of pathogens and *C*_*t*_ is the concentration of response cells at time *t*. Both infection and response to infection occur during consecutive small time intervals (*t* + Δ*t*). Within a time interval, the host is infected by *D*_*t* + Δ*t*_ new pathogens while pathogens present within the host multiply (*N*_*t* + Δ*t*_) and are killed by response cells (*K*_*t* + Δ*t*_). In the absence of infection, *M*_*t* + Δ*t*_ response cells reach the tissues while an extra-concentration (*G*_*t* + Δ*t*_) is recruited and removed (*S*_*t* + Δ*t*_) in case of infection. All concentrations are homogeneous Poisson processes: the number of events in time interval (*t* + Δ*t*) follows a Poisson distribution with associated specific rates that are described more specifically in the following section.

The symbol *D*_*t* + Δ*t*_ represents the concentration of pathogens effectively transmitted and inoculated to one host after contact with a number I of infective hosts, each infected with *B*^*i*^_*t*_ pathogens. It is governed by the equation:

$$D_{t+\Delta t}=\sum_{i}cv\beta^{i}B_{t}^{i}\text{ for }i=1,\;2,\;\cdots I,$$

where *c* = probability of contact between the host and an infective host, ν = the fraction of the infective dose actually inoculated by the host, and β^*i*^ = fraction of *B*^*i*^_*t*_ the infected host excrete during an effective contact. Stated otherwise, β *B*^*i*^_*t*_ is the infective dose released by an infected host and ν represents the host anti-infection resistance.

The concentration of pathogens resulting from reproduction (*N*_*t* + Δ*t*_) is controlled by their multiplication rate, here assumed to be logistic:

$$N_{t+\Delta t}\sim \text{Poisson}[\gamma \;B_{t}(1-B_{t}/K_{B})]$$

In the equation, the per-capita growth rate (γ) is a function of the ability of pathogens to multiply until they reached their maximum concentration (*K*_*B*_). This behavior has indeed been observed in well-mixed *in vitro* suspensions (Malka et al., 2010).

Concurrently to infection, response cells are activated to kill *K*_*t* + Δ*t*_ pathogens:

$$K_{t+\Delta t}\sim \text{Poisson}[\alpha C_{t}\;B_{t}\rho /(1+(\tau \alpha B_{t}))]$$

where α is the maximum kill rate, τ is the time necessary for the cell to capture and kill the pathogen, and ρ is a scaling parameter representing the relative level of resistance of the host with theoretical limits at 0 or 1. If ρ = 0, the host is not resistant at all and cannot recover. The level of resistance is maximum at ρ = 1. In the science of ecology, the equation is called the Holling Type 2 functional response that describes the average feeding rate of a predator (here, a response cell) when the predator spends some time searching for a prey (here, a pathogen) and some time, exclusive of searching, processing each captured prey (Holling, 1959).

In the second part of equation [1], *M*_*t* + Δ*t*_ is the normal concentration of response cells in the tissue environment:

$$M_{t+\Delta t}\sim \text{Poisson}[\omega (C_{1}-C_{t})]$$

where ω is the natural rate at which cells are recruited and removed due to death or migration.

When pathogens are present, an extra-concentration of cells is recruited:

$$G_{t+\Delta t}\sim \text{Poisson}[\mu B_{t}C_{t}/(K_{m}+B_{t})]$$

where μ is the maximum rate of recruitment and *K*_*m*_ is the half-saturation constant. Then, if *B*_*t*_ is low, *G*_*t* + Δ*t*_ ~ Poisson [μ C_*t*_/K_*m*_], and reaches *G*_*t* + Δ*t*_ ~ Poisson [μ C_*t*_] when *B*_*t*_ is high.

The symbol *S*_*t* + Δ*t*_ represents the extra-removal of response cells after infection:

$$S_{t+\Delta t}\sim \text{Poisson}[\alpha C_{t}B_{t}\rho /(\theta (1+\tau \alpha B_{t}))]$$

The rate is called the numerical response rate (change in predator concentration as a function of change in prey concentration) and corresponds to the above Holling type II functional rate.

A response cell kills on average θ pathogenic cells before removal.

### System of equations for performance

Only the effects of pathogen and cells concentrations on hosts performances are considered. All other effects, such as resource intake, management or age are assumed fixed. Then,

(2) $$P_{t}=P_{1}-[B_{t}L_{B}(1-\lambda_{b})+C_{t}L_{c}(1-\lambda_{c})]$$

where *P*_*t*_ is the performance of the host in the presence of *B*_*t*_ pathogens and *C*_*t*_ response cells. The parameters *L*_*B*_ and *L*_*C*_ are the maximum performance lost per pathogen (virulence) and response cell, respectively. The parameters λ_*b*_ and λ_*c*_ are scaling parameters representing the relative ability of the host to tolerate damages caused by pathogens and immune cells. If λ_*b*_ = λ_*c*_ = 1, the host is completely tolerant and produces at the initial level (*t* = 1). If λ_*b*_ = λ_*c*_ = 0, the host is not tolerant at all. Although unrealistic (Can an animal be totally tolerant or un-tolerant?), the scaling parameters set the limits for *P*_*t*_ with a maximum at *P*_1_ and a minimum at *P*_1_−B_*t*_ L_*b*_−C_*t*_ *L*_*C*_.

Resistance and tolerance are associated with a redistribution of resources away from performance:

$$P_{1}=P^{\text{Max}}(1-\rho c_{p}-\lambda_{b}c_{b}-\lambda_{c}c_{c})$$

where *P*^Max^ is the maximal level of performance reached when levels of resistance and tolerance are null (ρ = λ_*b*_ = λ_*c*_ = 0). The parameter *c*_ρ_ is the relative costs of resistance while *c*_*b*_ and *c*_*c*_ are the relative costs of tolerance to pathogens (direct) and response cells (indirect).

### Values for the parameters

Values for parameters describing a healthy and early inflammatory response to infection are from studies on *E. coli* bovine mastitis (Table 1). Baseline values insure a healthy response such that pathogens are cleared and hosts return to pre-infection equilibrium. For the simulation, endemics start in a population of 50 susceptible hosts in which 2 are infected with concentrations of cells and bacteria close to 10^7^cells/μL, and 10^6^ bacteria/μL, respectively. The value 10^6^ bacteria/μL is the highest concentration observed in neutropenic cows (Rainard and Riollet, 2003) and 10^7^ cells/μL is the highest somatic cells concentration observed in a field survey of mastitis in Belgium (Detilleux et al., 2012). In non-infected hosts, concentrations of response cells (*C*_1_) are normally distributed with mean of 100 cells/μL and standard deviation of five cells/μL (Djabri et al., 2002). Once inside the hosts, bacteria grow at a rate of one new pathogen per hour and response cells migrate to the site of infection with a maximum migration rate of 2 μL/bacteria/h (Detilleux et al., 2006). The time for a response cell to capture and kill the pathogen and the concentration of bacteria killed per cell were set at 1 h (Adinolfi and Bonventre, 1988) and 10 bacteria (Nagl et al., 2002), respectively. The Holling Type II kill rate is 0.005/μL/bacteria/h (Detilleux et al., 2006). This means that as few as 0.005 bacteria are killed per cell and per h when *B*_*t*_ is small, and up to five bacteria are killed when *B*_*t*_ is high.

**Table 1:** **Symbol, signification and values of the parameters**.

**Symbol**	**Signification**	**Values**
**PARAMETERS WITH THE SAME VALUES IN ALL SIMULATIONS**		
*K*_*B*_	Maximum concentration of pathogens	10^6^/μL
*K*_*C*_	Maximum concentration of response cells	10^7^/μL
*P*^Max^	Maximum performance	100 units
γ	Pathogen logistic growth rate	1 pathogen/μL/h
τ	Time for a response cell to capture and kill pathogens	1 h/cell
θ	Pathogen concentration killed per response cell	10 pathogens/cell
*c*	Contact rate between hosts	0.1/h
**PARAMETERS FOR THE DIFFERENT RESPONSE SCENARIOS**		
*K*_*M*_	Pathogen concentration such that response cells reach the infection site in 1 time unit	
	Healthy response (scenario A)	10 cells/μL
	Recurrent infection (scenario B)	10000 cells/μL
α	Pathogen clearance rate	
	Healthy response (scenario A)	0.005 pathogen/cell/h
	Persistent infectious response (scenario C)	0 pathogen/cell/h
ω	Recruitment/elimination rate of response cells during health	
	Healthy response (scenario A)	0.5 cells/h
	Persistent non-infectious response (scenario D)	0.01 cells/h
μ	Extra-recruitment rate of response cells during infection	
	Healthy response (scenario A)	2 cells/μL/h
	Immuno-depression (scenario E)	0 cells/μL/h
**PARAMETERS WITH UNIFORM DISTRIBUTIONS**		
β	Infectiousness	U[0; 0.01]
*L*_*C*_	Loss associated with each response cell	U[0; 25/*K*_*C*_]
*L*_*B*_	Loss associated with each pathogen	U[0; 25/*K*_*B*_]
*c*_ρ_, *c*_*b*_, *c*_*c*_	Resistance, direct and indirect tolerance costs	U[0; 0.1]
ν	Resistance to infection	
	Low	U[0; 0.001]
	Average	U[0; 0.01]
	High	U[0.009; 0.01]
ρ	Resistance to disease	
λ_*b*_, λ_*c*_	Direct and indirect tolerances	
	Low	U[0; 0.1]
	Average	U[0; 1]
	High	U[0.9; 1]

Outcome of the inflammatory response is not always health. To determine whether the model could reflect such reality, scenarios for the inflammatory response, other than the healthy response (scenario A), were tested by modifying the values of the parameters (Kumar et al., 2004). In scenario B, response cells are not recruited rapidly to the site of infection, pathogens cannot be completely eliminated and the infection is recurrent. In scenario C, infection is persistent and infectious when response cells and pathogens concentrations are high; it is persistent and non-infectious when pathogens are cleared but response cells concentrations are high (scenario D). The last scenario (scenario E), severe immunodeficiency, occurs when pathogens multiplied up to saturation with no activation of response cells.

Without information in the literature, values for the rates in equations for performance were drawn from uniform distributions. A convenient value of 100 units was given to *P*^Max^. Individual levels in resistance and tolerance were drawn from distributions with different extreme values to have low (U[0, 0.1]), average (U[0, 1]), or high (U[0.9, 1]) levels. The maximum part of *P*_1_ available to resistance and tolerance was set at *P*^Max^/2. Individual tolerance and resistance costs were drawn from U[0, 0.1]. Highest direct (*L*_*b*_) and indirect (*L*_*C*_) loss associated with each pathogen were set at 25 × 10^−6^ units of performance lost per pathogen present, and 25 × 10^−7^ units of performance lost per response cell. The values for *L*_*B*_ and *L*_*C*_ were chosen to insure that *P*_*t*_ remains positive when costs and cell and pathogen concentrations are highest.

### Computations

All computations were done on SAS 9.1. Simulation steps were executed until *t* reaches 100 time-units or the disease dies out (= one cycle) and repeated over 1000 cycles. At the end of all cycles, individual performance (*P*_*t*_) and concentrations of host cells (*C*_*t*_) and pathogens (*B*_*t*_) were expressed as the percentages of their maxima (*P*^Max^, *K*_*C*_, and *K*_*B*_, respectively), averaged over all animals and all replications, and plotted across time. Similarly, the number of infected hosts (*I*_*t*_) was expressed as the percentage of the total number of hosts in the population (50) and averaged over all replications. To sum up, area under the curves of *P*_*t*_ (AUC_*P*_) and *I*_*t*_ (AUC_*I*_) were computed for *t* = 1–100 with the trapezoidal rule. Least-squares means of the AUCs were computed for high, average and low levels of tolerance and resistance.

## Results

This section starts with results about the ability of the model to simulate different scenarios of response to infection, at the animal and population levels. It follows by the effects of different levels of resistance and tolerance on a healthy response.

### Scenarios of response to infection at the animal level

**Figure 1:**
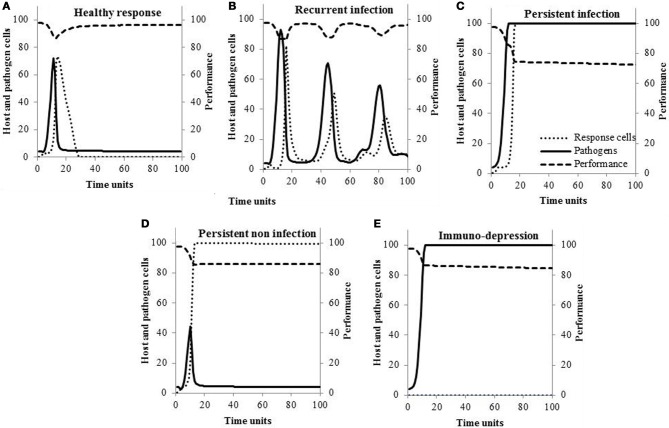
**Typical changes in the within-host concentrations of pathogens, response cells, and hosts performance according to the scenario of response to infection**.

Typical within-host curves are shown in Figure 1 for the five different scenarios of response to infection. The concentrations of pathogens increase within 20 time units and are followed by an increase in response cells. If the response is healthy (scenario A), pathogens are killed efficiently by the response cells, their concentrations decrease, and host performance returns to pre-infection values. This is contrary to what is observed when pathogens susceptibility to the response cells is null (Figure 1C): concentration of response cells reaches high values but pathogens cannot be cleared (scenario C). In Figure 1B, the increase in response cells is delayed so pathogens are not completely eliminated. Then, the infection is recurrent and associated with episodes of performance losses (scenario B). If the response is persistent and non-infectious (scenario D), the concentration of response cells remains elevated even though pathogens are killed. In the last scenario (Figure 1E), response cells are not activated and pathogens grow to saturation (scenario E).

### Scenarios of response to infection at the population level

**Figure 2:**
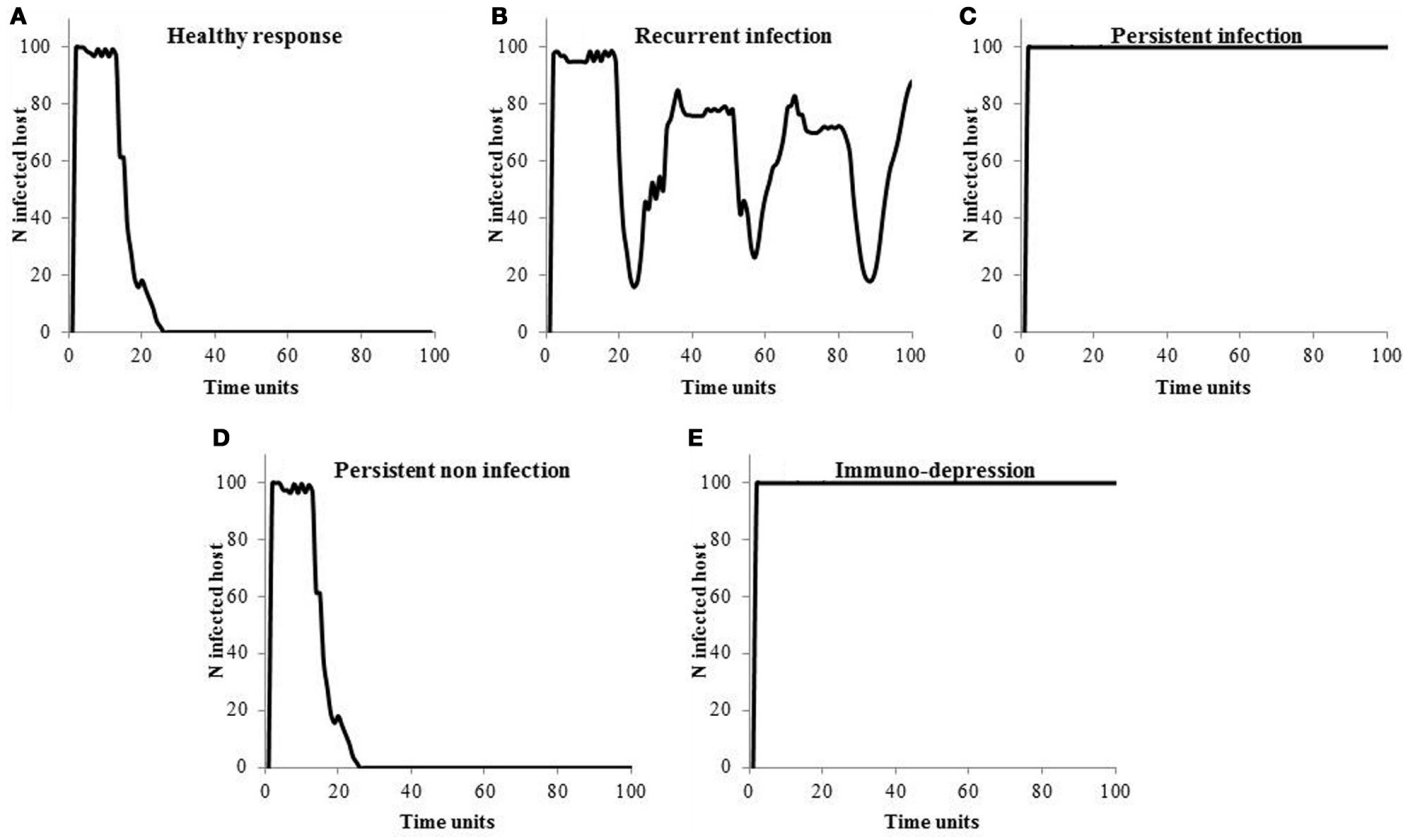
**Size of the endemics according to the scenario of response to infection**.

Figure 2 shows how the infection spreads in the population. If hosts are able to get rid of the infection (Figures 2A,D), the endemics dies out. If the infection is recurring within the host, so do the endemics at the population level (Figure 2B). In case of persistent infectious response and immune-depression (Figures 2C,E), hosts all become infected.

### Resistance and tolerance on within-host dynamics

**Figure 3:**
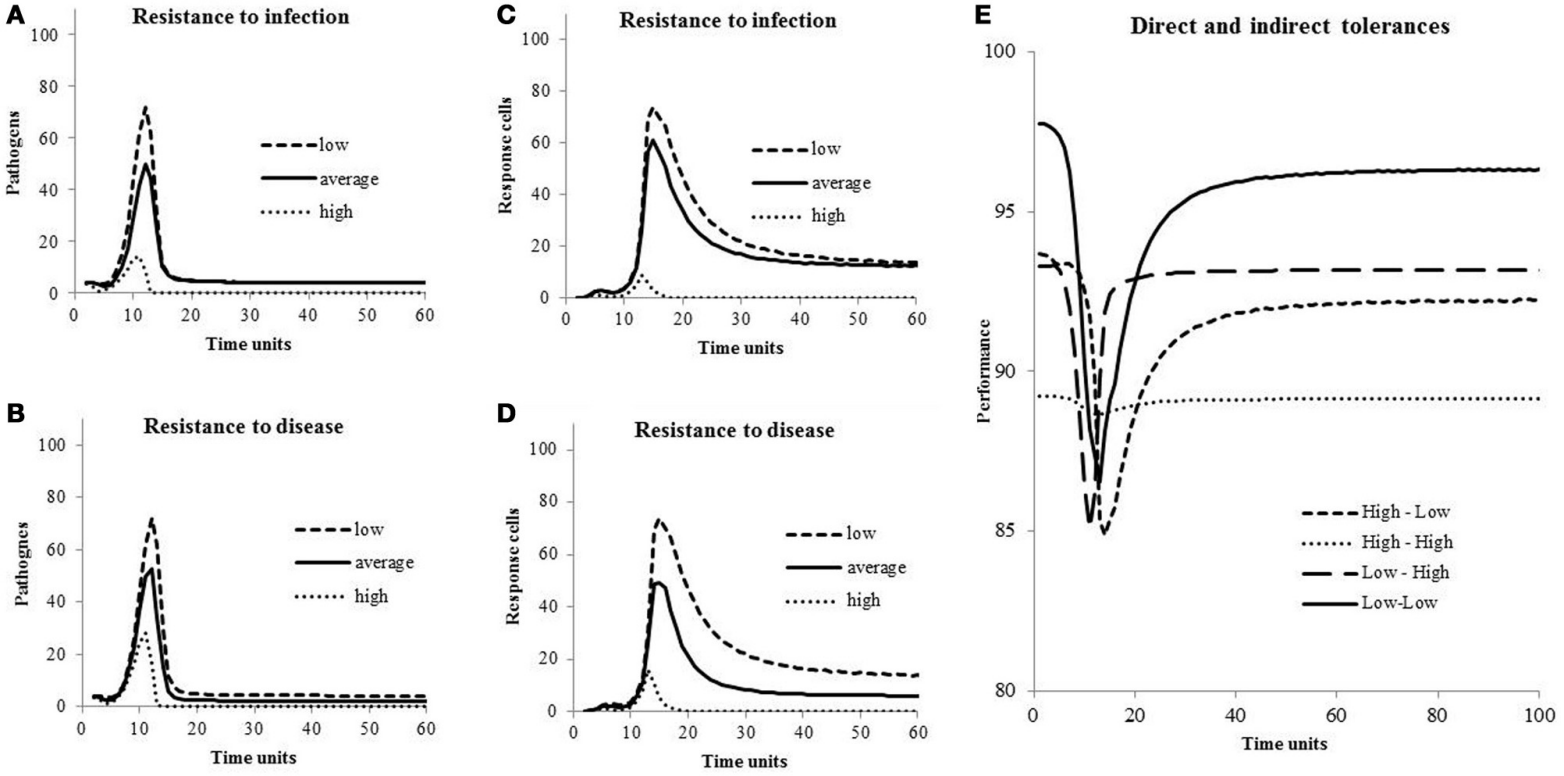
**Concentrations of pathogens and response cells for different levels of resistance to infection and to disease, and host performances for different levels of direct and indirect tolerances**. Values of the parameters are in Table 1.

In Figure 3, concentrations of pathogens (*B*_*t*_) during an episode of infection are shown for different levels of resistance to infection, concentrations of response cells (*C*_*t*_) are shown for different levels of resistance to disease, and host performances (*P*_*t*_) are shown for different levels of direct and indirect tolerance, all for hosts with a healthy response to infection. Peak of pathogen concentrations are high when both levels of resistance are low (Figures 3A,B). Similarly, concentrations of response cells necessary to fight pathogens increase when cell levels of resistance to disease and to infection move from high to low (Figures 3C,D). Performances decreased during the response to infection (Figure 3E) unless the host is highly tolerant to damage associated with both pathogens and response cells (line “High-High”). When both direct and indirect tolerance levels are lowest (line “Low-Low”), the loss during the period of infection is the highest with *P*_*t*_ going from 98% at *t* = 0 to 86% at *t* = 13 time-units but the loss is the lowest over the period from *t* = 0 to *t* = 100 time-units. Indeed, the loss from *t* = 0 to *t* = 100 time-units varies from 4.5% of the maximum performance (*P*_Max_) if the host is not tolerant (line “Low-Low”) to 10.9% if it is highly tolerant (line “High-High”). It is 7.2% and 8.3% if the host is tolerant to direct (line “High-Low”) or indirect (line “Low-High”) damages, respectively.

### Resistance and tolerance at population level

**Figure 4:**
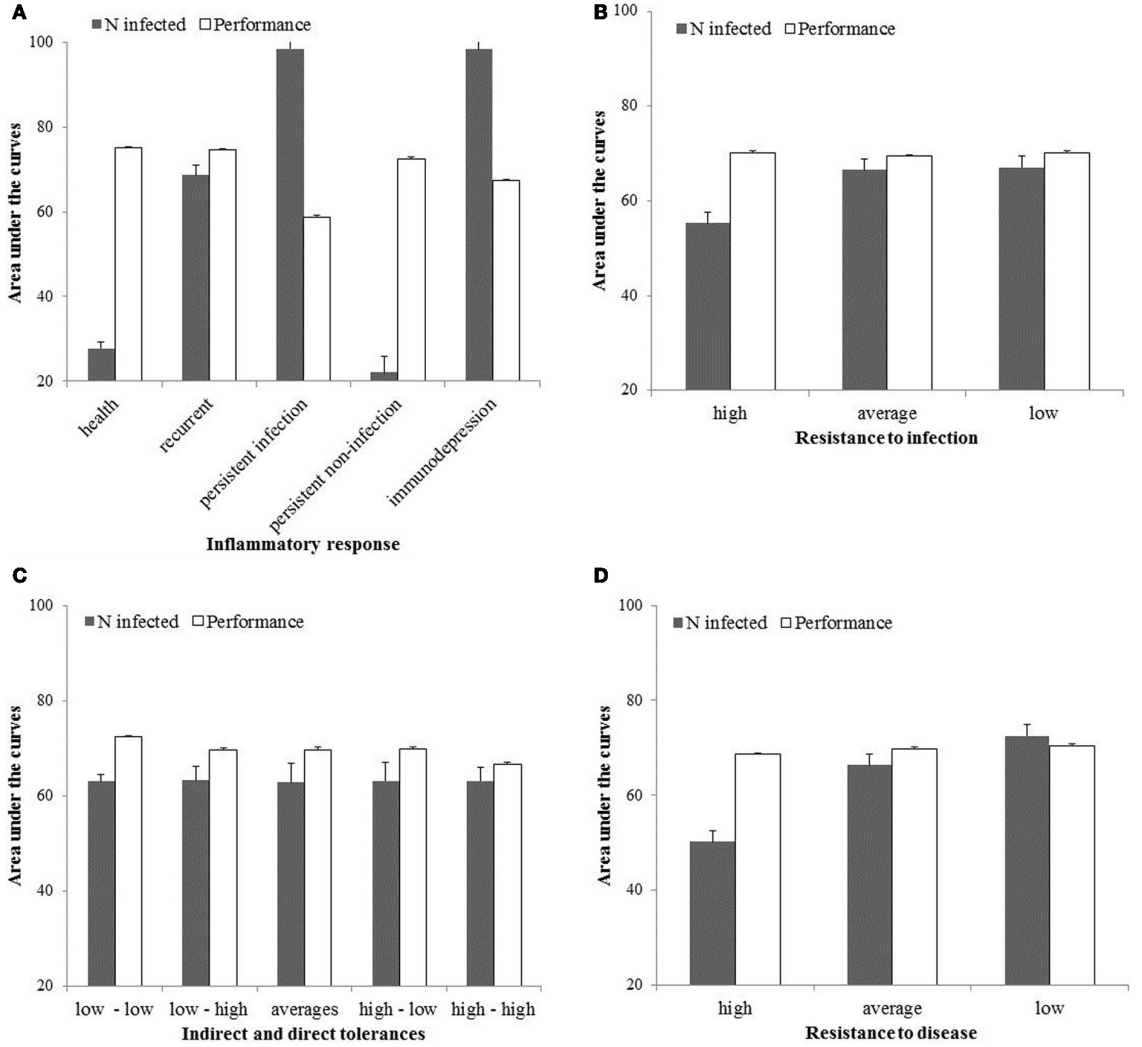
**Area under the curves for the host performances and number of infected in the population for the first 100 time-units and expressed as the percentages of their respective maxima**.

The area under the curves of performances (AUC_*P*_) and number of infected hosts (AUC_*I*_) are shown in Figure 4 for high, average and low levels of resistance to infection and disease, and for high, average and low levels of direct and indirect tolerance. The AUC_*P*_ is the highest (most favourable) when hosts mount a healthy response to infection, are highly resistant to disease and infection, and not tolerant to both direct and indirect damages associated with the infection. It is the lowest when hosts are persistently infected, not resistant to disease and infection, and highly tolerant to both direct and indirect damages associated with the infection. The AUC_*I*_ is the highest (most favourable) when hosts are immunodepressed or persistently infected with low levels of resistance to infection and disease. It is the lowest when hosts mount a healthy or persistent response to infection and are highly resistant to infection and disease. Indirect and direct levels of tolerance have no effect on AUC_*I*_.

## Discussion

A mathematical model is proposed to quantify the effects of resistance and tolerance on the spread of an infectious disease (here, *E. coli* mastitis) and on animal performances within a closed population. Such theoretical studies are necessary because resistance and tolerance are difficult to be measured in field studies. Actually, resistance is typically assessed by measuring infection intensity, i.e., bacteriological cultures in the case of bovine mastitis. However, such information is often lacking because it is time-consuming and costly to obtain. Indirect measures of infection intensities (e.g., somatic cell counts, conductibility, and clinical signs) have also been proposed but their accuracy in evaluating the udder bacteriological status is low. Even when the information is available, different intensities may be the fact of different levels of resistance but also of different chances of encountering pathogens. Indeed, a susceptible animal in a population free from the pathogen has no opportunity to get infected and may be erroneously classified as resistant. When infection intensity is available, one can also measure tolerance as the slope of a regression of host performance against infection intensity. But this measure does not distinguish between direct and indirect tolerances. Costs of resistance and tolerance are even more difficult to quantify in practice since their measures necessitate evaluating hosts performances in pathogen-free environments (Råberg et al., 2009).

If they are necessary, models should also adequately reflect reality. Although simple, the model proposed here allows simulating scenarios that have all been observed in animals. For example, changes in pathogens and cell concentrations depicted in Figure 1A (scenario A) were previously described in cows experimentally infected with different *E. coli* doses (Vangroenweghe et al., 2004). Burvenich et al. (2003) showed phagocytes with low killing ability (Figure 1C) cannot sustain an effective elimination of the pathogen and the resolution of *E. coli* mastitis (scenario C). It is also known that cows suffering from the leukocyte adhesion deficiency syndrome (scenario E) present persistent infection (Figure 1E) due to the lack of molecules necessary for neutrophils to migrate out of the blood stream toward the site of infection (van Garderen et al., 1994). As a final example, Hill (1981) showed infections can persist and lead to recurrent clinical mastitis (Figure 1B) when the speed at which neutrophils are mobilized in the gland is low (scenario B).

Within the range of selected values (Table 1), the model suggests breeding should be for animals mounting a healthy response to infection and highly resistant to disease or infection. Then, performances at the population level will be the highest and endemics the smallest (Figure 4). In this particular situation and if resistance is independent of tolerance, improving tolerance should not be considered as a selection objective because it is redundant to resistance: an already resistant host will not get infected or diseased so energy is not necessary to tolerate damages linked with infection. Note however several mechanisms have been shown to influence both resistance and tolerance (Shinzawa et al., 2009; Ayres and Schneider, 2012) so selection for resistance can result in a correlated response in tolerance. Other potential factors that may influence the decision of whether improvement of resistance is beneficial over improvement of tolerance have been ignored in this model. These may include host-pathogen co-evolution (e.g., Roy and Kirchner, 2000), infection-induced reduction in resource intake (e.g., Sandberg et al., 2006) or different shapes of cost functions associated with resistance and tolerance (e.g., Restif and Koella, 2004).

Values for costs and effects of resistance/tolerance on host performances were chosen arbitrarily because no information was found in the literature. An exception is the experiment of Råberg et al. (2007, 2009) on laboratory mice inoculated with the rodent malaria parasite *P. Chabaudi*. They observed approximately 10% decrease in weight and red blood cell density per 3 μL^−1^ × 10^6^ parasites. But even though they are based upon arbitrary values, effects shown in Figure 3 are relatively coherent: Pathogen and response cells concentrations increase when resistance to both infection and disease decreases (Figures 3A–D). During an episode of infection, losses in performance are highest in hosts not tolerant to damage associated with the presence of pathogens and the response to infection (Figure 3E, line “Low-Low”). But, on a period of *t* = 1–100 time-units, the loss is the smallest because not tolerant hosts have set little resources away from performances and return to higher performance levels after the episode of infection. Note this loss is around 5% of the maximum performance and corresponds, luckily, to the loss in milk production associated with clinical mastitis case at the lactation level (meta-analysis of Seegers et al., 2003).

Another drawback of the model is that hosts in the population all present one particular scenario of response to infection (Figure 2) although studies suggest a genetic influence on the response to infection (Davies et al., 2009). For example, in Holsteins, heritabilities have been reported for neutrophils migration (0.2–0.5), for neutrophils phagocytosis (0.3–0.7), for cellular-mediated adaptive response (0.16) and for antibody-mediated adaptive response (0.2–0.4) (Detilleux et al., 1994; Thompson-Crispi et al., 2012). To account for these differences, the model could easily be made more realistic by simulating different scenarios for different individuals and also for different periods in the same individual.

No link was considered between scenario of response to infection and costs of resistance/tolerance, considering that costs were constitutive and allocated in a pathogen-free environment (Rohr et al., 2010). But one may argue that animals with more resources for defense-related pathways, rather than performance, will preferentially mount a healthy rather than another type of response. They may defend themselves by over-expressing specific defense pathways in a temporal and spatial manner rather than wide-ranging constitutive mechanisms (Medzhitov et al., 2012). Conversely, too many resources could lead to excessive immune response and immuno-pathology (Colditz, 2002). So, the question remains whether selection objective should be for animals with constitutive or inducible resistance/tolerance.

In conclusion, the model is useful in shedding some light on the complex interactions between resistance/tolerance and performance but needs realistic values to better grasp the processes. To improve it, we are planning a small explorative study, funded by the European research group EADGENE_S, to measure animal levels of resistance/tolerance to bovine mastitis in herds located in Wallonia. Resistance will be measured by the number of bacteria colony forming units in milk of cows located in herds in which cows' opportunity to get infected is measureable (Detilleux et al., 2012). Direct and indirect tolerances will be quantified with structural equation models linking somatic cell counts, colony forming units and milk production. Hopefully, this will give us some clues on how to choose our selection objectives and improve the health of animals in an economic environment.

### Conflict of interest statement

The author declares that the research was conducted in the absence of any commercial or financial relationships that could be construed as a potential conflict of interest.
